# Dosimetric evaluation of a 320 detector row CT scanner unit

**DOI:** 10.2478/v10019-010-0049-1

**Published:** 2010-11-25

**Authors:** Mario de Denaro, Paola Bregant

**Affiliations:** S.C. di Fisica Sanitaria, A.O.U. “Ospedali Riuniti” di Trieste, Trieste, Italy

**Keywords:** multislice CT, pencil chamber, computed tomography dose index, dose profile, Gafchromic film

## Abstract

**Background:**

The technologic improvements in Multislice scanners include the increment in the X-ray beam width. Some new CT scanners are equipped with a 320 detector row which allows a longitudinal coverage of 160 mm and a total of 640 slices for a single rotation. When such parameters are used the length of the traditional pencil chamber (10 cm) is no more appropriate to measure the standard weighted computed tomography dose index (CTDI_w_) value.

**Materials and methods.:**

Dosimetric measurements were performed on a 640 slices Toshiba Aquilion One CT scanner using common instrumentation available in Medical Physics Departments.

**Results:**

For the measurements in air, two different ionization chambers were completely exposed to the beam. Dosimeters showed an acceptable agreement in the measurements. To evaluate the actual shape of the dose profile strips of Gafchromic XRQA film were used. Films were previously calibrated on site. From the graphic response of the scanned film it is possible to evaluate the full width at half maximum (FWHM) of the dose profile which represent the actual beam width.

**Conclusions:**

Computed Tomography Dose Index (CTDI) and Dose Length Product (DLP) need to be changed when the beam width of the CT scanner is over 100 mm. To perform dose evaluation with the conventional instrumentation, two parameters should be considered: the average absorbed dose and the actual beam width. To measure the average absorbed dose, the conventional ionization chamber can be used. For the measurement of the width of the dose profile, Gafchromic XRQA film seemed to be suitable.

## Introduction

The advent of multislice scanners technology^1^ leads to a continuous increment of the X-ray beam width along the cranio-caudal direction. The new CT scanner from Toshiba, *Aquilion One*, is equipped with the 320 detector rows each one 0.5 mm wide. This new technology allows a longitudinal coverage of 160 mm at rotation time of 0.35 seconds. The important change in dosimetric evaluation is needed to adapt the CT dosimetry metrics to the new standard.^2^–^5^ With a 160 mm beam width as an operating condition, the length of the traditional pencil chamber (10 cm) is no more appropriate to evaluate the standard computed tomography dose index (CTDI).^6^ Despite this problem the CTDI and the dose length product (DLP) values are still displayed on the scanner console. The problem for medical physicists, who must carry out quality assurance and dose optimization on the new CT equipment, is that suitable instrumentation is usually not available. In the present work we suggest some dosimetric measurements that can be carried out on a 320 row CT scanner unit, by means of conventional dosimetric instrumentation.

## Material and methods

**FIGURE 1.**
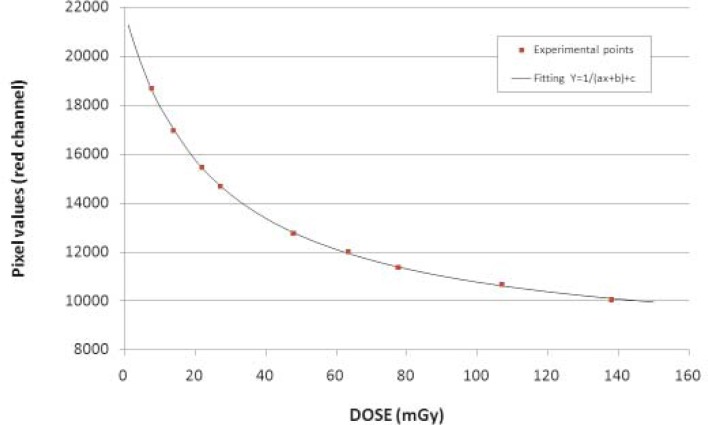
Fitting curve for the calibration of Gafchromic XRQA film.

Some measurements on a *640 slices Toshiba Aquilion One* CT scanner were carried out by means of common instrumentation typically employed at Medical Physics Departments. CT scanners providing 160 mm beam width allows to completely expose the 6cc ionization chamber of a Radcal Dosimeter, model 9015, commonly employed in the conventional radiodiagnostic measurement. To evaluate the average absorbed dose due to the primary beam, we compared the measurement carried out in air at the isocenter of the gantry, during a single rotation, exposing together the Radcal Dosimeter and the standard 10 cm long CT pencil chamber (WDCT 10, Wellhofer, Germany), connected to PMX-III electrometer. The average absorbed dose alone does not give information regarding the profile of the dose along the cranio-caudal direction (Z axis); it is a parameter to be considered to evaluate the shape and the actual width of the beam. To measure the dose profile free in air Gafchromic XRQA film was exposed to the primary beam during a single axial rotation. The film was previously calibrated exposing small pieces of Gafchromic (2 × 2 cm) to increasing doses; the Gafchromic films were put close to a reference ionization chamber. A calibration curve specific for the CT scanner beam quality was then obtained and it was used to convert the pixel values of the film into dose values (Figure 1).^7^

**FIGURE 2.**
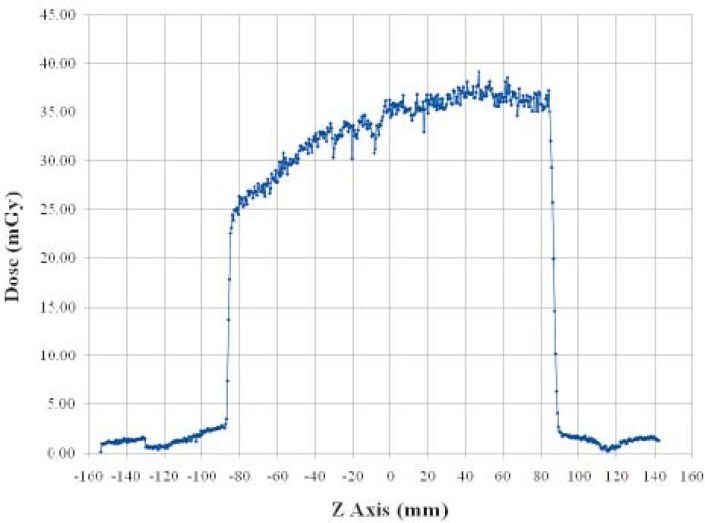
Dose profile measured by Gafchromic XRQA film.

The exposed film was then scanned by a regular scanner (Epson 1680 pro) using red-green-blue (RGB) modality at 48 bits of depth and spatial resolution of 72 dots per inch. Only the red component of the image was considered. The dose profile was obtained correcting the pixel values by the calibration curve (Figure 2).

**FIGURE 3.**
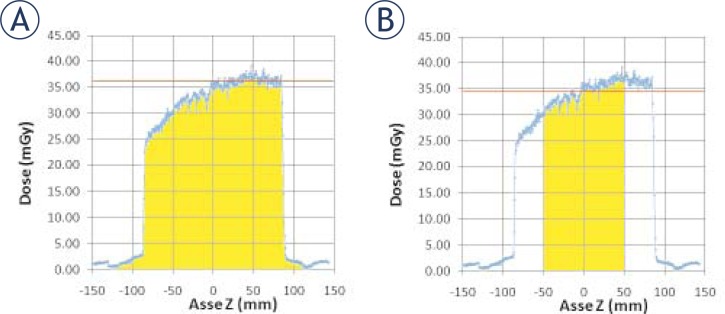
(A) Computed tomography dose index (CTDI)_air,300_. (B) CTDI_air, 100_.

From the graph the full width at half maximum (FWHM) of the dose profile can be evaluated. FWHM is a parameter which is related to the Z-axis geometric efficiency. CTDI_air,300_ (CTDI value using an integration interval of 300 mm) and CTDI_air,100_ (CTDI value using an integration interval of 100 mm) were evaluated and compared (Figure 3).^2^

**FIGURE 4.**
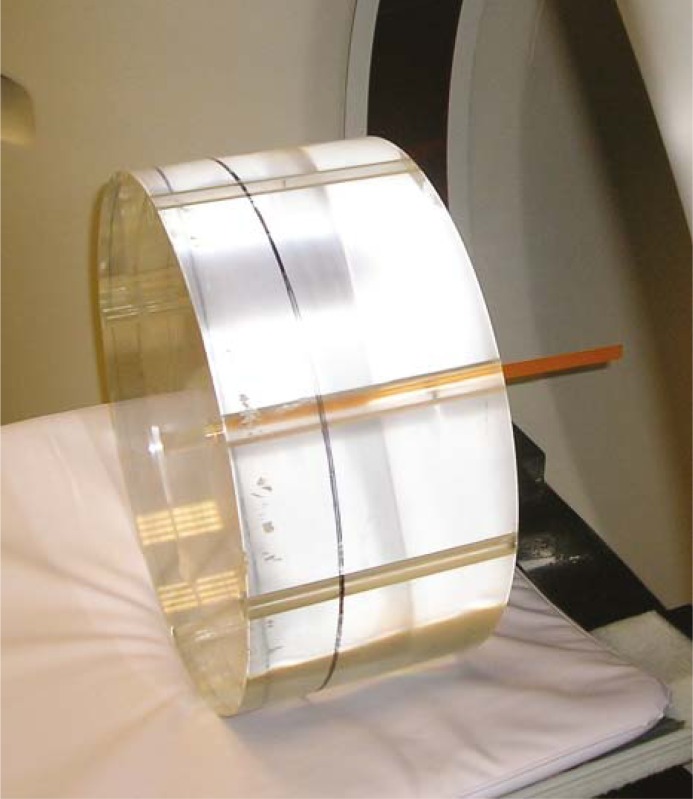
Strip of Gafchromic film inserted inside the hole of a poliymethyl methacrylate (PMMA) phantom.

**FIGURE 5.**
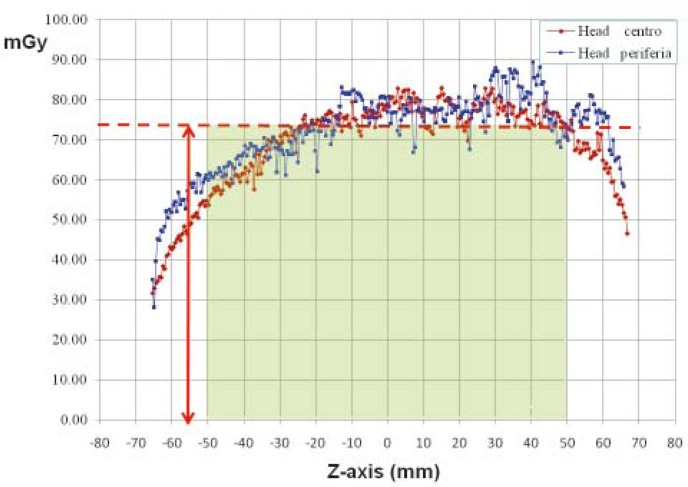
Average dose evaluation by Gafchromic film into the poliymethyl methacrylate (PMMA) phantom.

Finally, we put a strip of Gafchromic film in the holes of a standard CT dosimetric PMMA phantom (Figure 4) to evaluate the average absorbed dose inside the phantom (Figure 5).

## Results

The measurement performed by the Radcal dosimeter showed an acceptable agreement with the values obtained by the Wellhofer pencil chamber (Table 1). The comparison was meaningful because the 6 cc chamber of the Radcal dosimeter is completely exposed by the beam width.

To maintain the meaning of the CTDI, the integration interval for 160 mm beam width must be extended to a value of at least 300 mm.^2^ For the measurement of the weighted computed tomography dose index (CTDI_w_) a PMMA phantom is required, but at the moment it is very difficult to find and manage a PMMA phantom with a length of 300 mm. Moreover, also for the measurement of CTDI in air the length of the conventional pencil chamber (100 mm) is inappropriate. A way to perform dosimetric measurements using conventional instrumentation is to use the ionization chamber for the measurement of the average absorbed dose and Gafchromic film for the evaluation of the actual shape of the dose profile along z-axis. In Figure 1 the shape of the dose profile is plotted; an asymmetry appears evident in the profile due to the anode heel effect. Analysing the profile FWHM was calculated to be 171.8 mm pointing out an overbeaming of 11.8 mm. Z-axis geometric efficiency was also evaluated; it is defined by the International Electrotechnical Commission (IEC)^8^ as the ratio between the integral over the range subtended by detectors and total integral of the dose profile. The measured value of 95.4% showed for this parameter a low contribution of the penumbra effect.

In Figure 3 two different intervals of integration to evaluate the CTDI_air_ are presented; the value of CTDI_air,300_ was 36.7 mGy and the value of CTDI_air,100_ was 34.5 mGy. The result was as expected because the longer interval takes into account also the contribution due to the tails of the dose profile.

To obtain an estimation of the absorbed dose inside the PMMA phantom, a strip of Gafchromic film was inserted in the phantom holes. The resulting value of 74.7 mGy was compared with the value of 81.4 mGy displayed at the CT console as CTDI_vol_. The comparison showed a significant underestimation of the measured value probably due to the length of the PMMA phantom (15 cm) which is not long enough to simulate completely the effect of the scattered radiation.

## Discussion

Multislice CT scanners lead to a progressive increment in the X-ray beam width along z-axis. CTDI appears to be no more suitable to represent the main dosimetric quantity in CT dosimetry. Recent publications try to find new dose parameters more representative of the technological state of art.^9^

The conventional quality assurance and the dose assessment would require an upgrade in the conventional instrumentation, but new suitable dosimeters and phantoms are not yet available for most Medical Physics Department. Nevertheless, dosimetric parameters for a CT scanner with a beam width of 16 cm can be estimated by means of commonly available dosimetric instrumentation.

The average absorbed dose related to a single axial rotation can be measured by conventional ionization chambers, while the geometric dose distribution along a profile in the cranio-caudal direction (Z-axis) can be evaluated exposing Gafchromic film to the primary beam.

## Figures and Tables

**TABLE 1.** Absorbed dose evaluated with two different dosimeters

**Dosimeter**	**Dose FOV Head (mGy/mAs)**	**Dose FOV Body (mGy/mAs)**
**Wellhöfer pencil chamber (WDCT 10), with PMX-III**	0.257	0.225
**Radcal model 9015with 6cc chamber 10X5–6**	0.272	0.243

FOV, field-of-view; WDCT 10, Wellhöfer CT pencil chamber 10 cm long

## References

[1] Beslic S, Beslic N, Beslic S, Sofic A, Ibralic M, Karovic J (2010). Diagnostic imaging of traumatic pseudoaneurysm of the thoracic aorta. Radiol Oncol.

[2] Geleijns J, Salvadó Artells M, de Bruin PW, Matter R, Muramatsu Y, McNitt-Gray MF (2009). Computed tomography dose assessment for a 160 mm wide, 320 detector row, cone beam CT scanner. Phys Med Biol.

[3] (2008). The measurement, reporting, and management of radiation dose in CT.

[4] (2010). Comprehensive methodology for the evaluation of radiation dose in x-ray computed tomography.

[5] Rimondini A, Pozzi Mucelli R, De Denaro M, Bregant P, Dalla Palma L (2001). Evaluation of image quality and dose in renal colic: comparison of different spiral-CT. Eur Radiol.

[6] Brenner DJ (2005). Is it time to retire the CTDI for CT quality assurance and dose optimization?. Medical Physics.

[7] de Denaro M, Bregant P, Severgnini M, de Guarrini F (2007). In vivo dosimetry for estimation of effective doses in multislice CT coronary angiography. Medical Physics.

[8] 62B - Committee on Diagnostic Imaging Equipment (2002). IEC 60601-2-44. Particular requirements for the safety of X-ray equipment for computed tomography. Medical electrical equipment.

[9] 62B - Committee on Diagnostic Imaging Equipment (2009). IEC 60601-2-44. Particular requirements for the basic safety and essential performance of X-ray equipment for computed tomography. Medical electrical equipment.

